# Psychological Impact of Type of Breast Cancer Surgery: A National Cohort Study

**DOI:** 10.1007/s00268-022-06585-y

**Published:** 2022-05-10

**Authors:** Soo kyung Ahn, Sohee Oh, Jongjin Kim, Jung-Seok Choi, Ki-Tae Hwang

**Affiliations:** 1 Department of Surgery, Kangnam Sacred Heart Hospital, Hallym University College of Medicine, 1 Shingil-ro, Youngdeungpo-ku, Seoul, 07441 Republic of Korea; 2 Medical Research Collaborating Center, Seoul Metropolitan Government Seoul National University Boramae Medical Center, Seoul, 07061 Republic of Korea; 3 Department of Surgery, Seoul Metropolitan Government Seoul National University Boramae Medical Center, Seoul, Republic of Korea; 4 Department of Psychiatry, SMC, Seoul, Republic of Korea; 5 Department of Surgery, Seoul National University College of Medicine, Seoul Metropolitan Government Seoul National University Boramae Medical Center, 39, Boramae-Gil, Dongjak-gu, Seoul, 07061 Republic of Korea

## Abstract

**Background:**

The present study assessed the impact of different types of breast surgery on rates of psychological disorders in breast cancer patients.

**Methods:**

This nationwide cohort study, based on Korean Health Insurance Review and Assessment Service claims data, included 26,259 breast patients who underwent surgery from June 1, 2017, to December 31, 2018. Associations between the incidence of psychological disorders and variables were evaluated by time dependent Cox regression analyses.

**Results:**

Of the 26,259 patients, 9394 (35.8%) underwent total mastectomy (TM) and 16,865 (64.2%) underwent partial mastectomy (PM); of the former, 4056 (43.2%) underwent breast reconstruction surgery (RS). A total of 4685 patients (17.84%) were newly diagnosed with psychological disorders after surgery. Multivariable analysis showed that axillary lymph node dissection was significantly associated with increased rates of overall psychological disorders (*p* < 0.0001), depression (*p* = 0.0462), anxiety (*p* < 0.0001) and insomnia (*p* < 0.0001). The rates of overall psychological disorders (*p* = 0.0002) and insomnia (*p* = 0.01) were significantly lower in patients who underwent TM than PM. RS tended to associated with reduced rates of overall psychological disorders in patients who underwent TM. Subgroup analysis showed that, compared with PM, RS after TM significantly associated with a reduced incidence of overall psychological disorders and insomnia in younger patients (< 50 years) and those who underwent sentinel lymph node biopsy.

**Conclusion:**

In contrast to general belief, rates of overall psychological disorders and insomnia were lower in patients who underwent TM than PM. Moreover, RS after TM confers psychological benefit in younger patients with early stage breast cancer compared with PM.

**Supplementary Information:**

The online version contains supplementary material available at 10.1007/s00268-022-06585-y.

## Introduction

The survival rates of breast cancer patients have increased due to earlier detection and improvements in treatments. These developments have resulted in increases in the numbers of women surviving breast cancer. These women undergo a wide range of functional and emotional impairments. Breast cancer patients experience persistent psychological problems, including poor body image, lower quality of life (QOL) and varied emotional disorders [1]. Mastectomy without breast reconstruction has been reported to increase the severity of psychological disorders, such as depression and anxiety [2–4], as well as to increase the incidence of insomnia [5, 6], among patients with breast cancer. Asymmetry of the chest and scars after mastectomy can cause psychological damage, and symptoms of psychological disorders have been associated with poorer physical function [7] and higher mortality risk [8]. Moreover, depression was found to be strongly associated with mortality in younger patients with early stage breast cancer [9].

Studies have investigated differences in the incidence of psychological disorders between patients who have undergone total mastectomy (TM) and partial mastectomy (PM), as well as the effects of breast reconstructive surgery (RS) in these patients [10]. Although most studies have shown that the incidence of psychological disorders is lower following PM than TM [11–13], other studies have reported the opposite results [14, 15]. Similarly, although RS after mastectomy has been reported to reduce psychological damage associated with mastectomy in patients with breast cancer [16, 17], other studies have reported contradictory findings [18–20]. Because most of these studies were single-center studies involving a relatively small number of patients, it is necessary to compare the effects of TM and PM and to analyze the impact of RS on psychological problems in breast cancer patients using accurate and comprehensive big data.

The purpose of this study was to assess the impact of different types of breast surgery on the incidence of psychological disorders in breast cancer patients.

## Materials and methods

### Data collection

The Republic of Korea National Health Insurance (NHI) system is a public medical insurance system that enrolls most citizens in the country. In addition, the Korean government operates Medical Aids, which provide medical services to low-income people unable to pay for the NHI.

Medical providers are required to submit claims for reimbursement for medical procedures covered by the NHI. Health insurance Review and Assessment Service (HIRA) data were obtained from the payment claims generated during patient visits or inpatient admissions to medical institutions. This cohort study was based on the HIRA claims data and included all patients who were diagnosed with invasive breast cancer and underwent surgery from June 1, 2017, to December 31, 2018. The starting date was chosen because data about whether TM or PM was performed were recorded only after June 1, 2017. The study protocol was approved by the institutional review board of the Hallym University Kangnam Sacred Heart Hospital (IRN No.2019-02-002) and Seoul Metropolitan Government Seoul National University Boramae Medical Center (IRN No.07-2019-4).

HIRA data were subjected to data mining using procedure codes for TM with (N7138) and without (N7139) axillary node dissection (ALND), and for PM with (N7136) and without (N7137) ALND. The types of reconstruction method were also analyzed (N7140–N7150) based on procedure codes.

Ascertainment of psychological disorder.

Because this study was designed to test the effect of the type of breast surgery on newly occurring psychological disorders, individuals with previous psychological disorders prior to the date of breast cancer diagnosis were excluded. All diagnoses in HIRA are coded according to the 10th Swedish revision of the International Classification of Disease (ICD), with depression coded as ICD10: F32-F33, anxiety disorder as ICD10: F40-F41, and insomnia as ICD10: F51; G47. Overall psychological disorder defined as if any of the three mental disorder occurred (Supp. Figure 1).

### Statistical analysis

Continuous variables were expressed as mean ± standard deviation (SD), or median (interquartile range [IQR]) and categorical variables as frequencies and percentages. Continuous variables were compared by two sample t-tests, analysis of variance (ANOVA), Mann–Whitney tests, or Kruskal–Wallis tests, as appropriate; whereas categorical variables were compared by Chi-square tests or Fisher’s exact tests, as appropriate.

The cumulative rates of overall psychological disorders, depression, anxiety, and insomnia were estimated using the Kaplan–Meier method and compared using log-rank tests. Risk factors associated with the overall incidence of psychological disorders were evaluated by univariable and multivariable time-dependent Cox regression analyses. The model included age, type of insurance, types of breast surgery, and axillary surgery. Additionally, time-dependent Cox regression analyses were performed in patients subgrouped by age and axillary surgery type. All statistical analyses were performed with SAS Enterprise Guide version 6.1 (SAS Institute Inc., Cary, NC, USA) software, with to *p *< 0.05 defined as statistically significant.

## Results

**Fig. 1:**
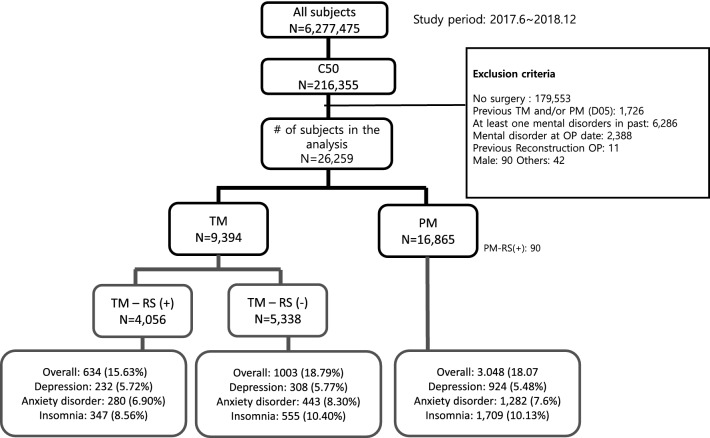
Patient selection.

A total of 26,259 patients diagnosed with invasive breast cancer and undergoing surgery between June 1, 2017, and December 31, 2018, were analyzed (Fig. 1). Median patient age at breast cancer diagnosis was 52.45 ± 11.08 years, and median follow-up time was 194 days [IQR 74–334 days]. Of the 26,259 patients, 9394 (35.8%) underwent TM and 16,865 (64.2%) underwent PM. Of the patients who underwent TM, 4056 (43.2%) underwent RS, with implant insertion being the most frequent reconstruction method (72.83%).

Among them, 4685 (17.84%) were newly diagnosed with psychological disorders after breast cancer surgery. Median time from surgery to diagnosis of a psychological disorder was 67 days (IQR 32–150 days). A total of 2611 patients (9.94%) were diagnosed with insomnia, 2005 (7.64%) with an anxiety and 1464 (5.58%) with depression (Table 1).

**Table 1:** Demographic and clinical characteristics of the study population

*N*	Total	TM (*N *= 9394)		PM	*P* value
		TM-RS(+)	TM-RS(-)		
	26,259	*N *= 4056	*N *= 5,338	*N *= 16,865	
Age, years	52.44 ± 11.07	46.56 ± 8.17	56.86 ± 12.26	52.46 ± 10.62	< 0.0001
< 40	2672 (10.18%)	781 (19.26%)	364 (6.82)	1527 (9.05%)	< 0.0001
40–49	9117 (34.72%)	1947 (48.00%)	1319 (24.71%)	5851 (34.69%)	
50–59	7935 (30.22%)	1050 (25.89%)	1577 (29.54%)	5308 (31.47%)	
≥ 60	6535 (24.89%)	278 (6.85%)	2078 (38.93%)	4179 (24.78%)	
Insurance type					< 0.0001
Health insurance	25565 (97.36%)	4016 (99.01%)	5028 (94.19%)	16521 (97.96%)	
Medical Aid	694 (2.64%)	40 (0.99%)	310 (5.81%)	344 (2.04%)	
Psychological disorder	4685 (17.84%)	634 (15.63%)	1003 (18.79%)	3048 (18.07%)	0.0002
Depression	1464 (5.58%)	232 (5.72%)	308 (5.77%)	924 (5.48%)	0.6559
Anxiety disorder	2005 (7.64%)	280 (6.90%)	443 (8.30%)	1282 (7.60%)	0.0399
Insomnia	2611 (9.94%)	347 (8.56%)	555 (10.40%)	1709 (10.13%)	0.0049
Reconstruction	4146 (15.79%)	4056		90	
LD flap w/o implant	240 (0.91%)	233 (5.74%)		7 (0.04%)	
TRAM w/o implant	892 (3.40%)	869 (21.43%)		23 (0.14%)	
Implant	3014 (11.48%)	2954 (72.83%)		60 (0.36%)	
Axillar surgery					< 0.0001
SLNBx	19398 (73.87%)	2854 (70.36%)	2799 (52.44%)	13745 (81.50%)	
ALND	6861 (26.13%)	1202 (29.64%)	2539 (47.56%)	3120 (18.50%)	

*TM* total mastectomy, *RS* reconstructive surgery, *PM* partial mastectomy, *LD* latissimus dorsi, *TRAM*, transverse rectus abdominal muscle, *SLNBx* sentinel lymph node biopsy, *ALND* axillary lymph node dissection

### Factors associated with psychological disorders

**Fig. 2:**
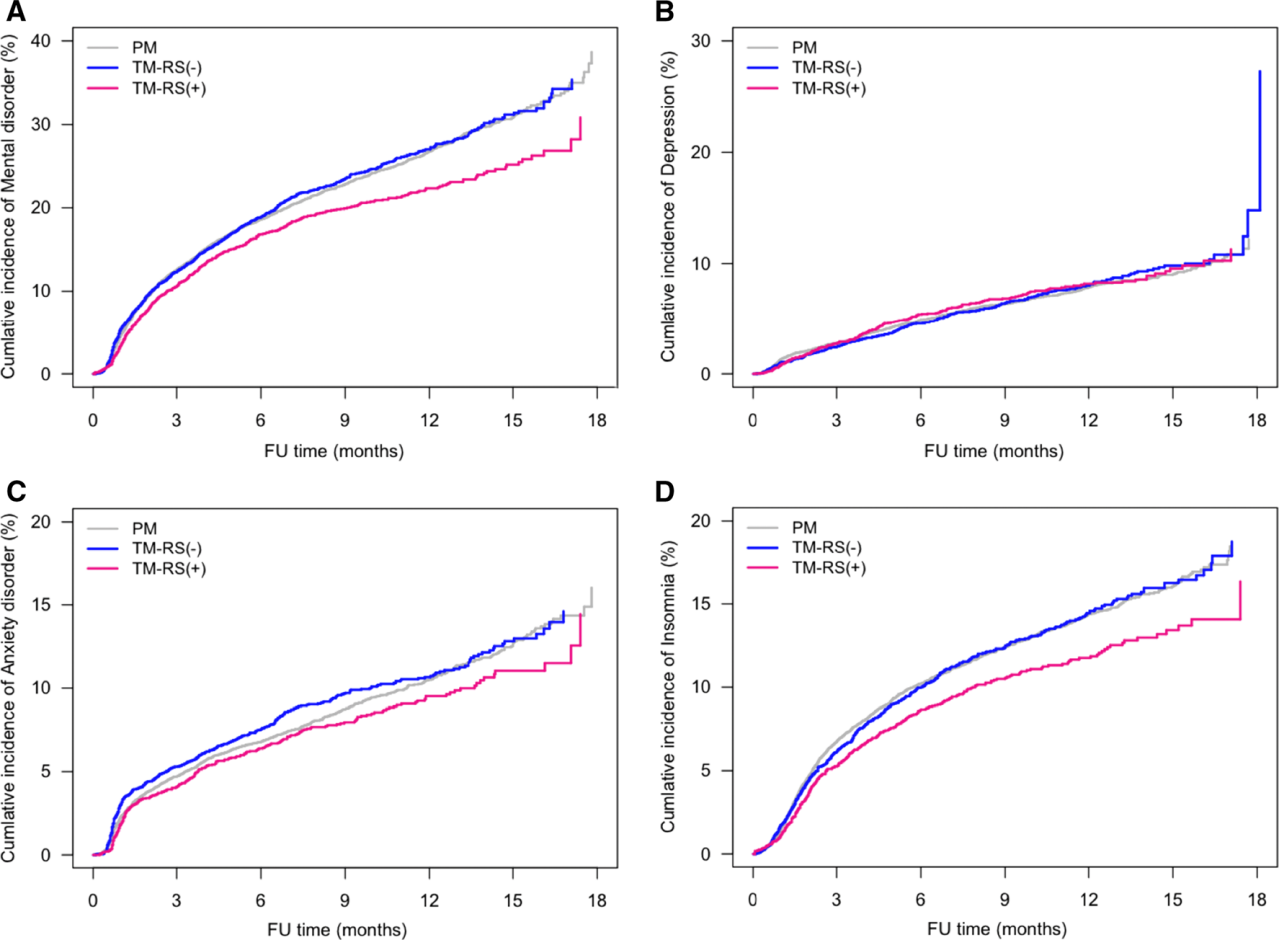
Kaplan–Meier analysis of the incidence of (**a**) overall psychological disorders, **b** depression, **c** anxiety, and **d** insomnia in patients who underwent TM plus RS, TM alone, and PM alone.

Factors associated with the development of psychological disorders, such as age, type of insurance, type of breast surgery and type of axillary surgery, were analyzed by time dependent Cox regression analysis (Table 2). Univariable analysis showed that participation in Medical Aid insurance (HR 1.185, *p* = 0.042) and axillary lymph node dissection (HR 1.305. *p* < 0.0001) were associated with significantly higher rates of overall psychological disorders. Compared with sentinel lymph node biopsy (SLNBx), ALND was associated with significantly higher rates of overall psychological disorders (HR 1.305, *p *< 0.0001), depression (HR 1.131, *p *= 0.0312), anxiety (HR 1.440, *p* < 0.0001) and insomnia (HR 1.203, *p* < 0.0001). Univariable analysis showed that rates of psychological disorders did not differ significantly in patients who underwent PM or TM, but that, compared with patients who underwent PM, TM plus RS was associated with significantly lower rates of overall psychological disorders (HR 0.847, *p* = 0.0001) and insomnia (HR 0.832, *p* = 0.0018). Rates of overall psychological disorders (HR 0.836, *p* = 0.0004) and insomnia (HR 0.835, *p* = 0.00086) were also significantly lower in patients who underwent TM plus RS than who underwent TM without RS (Fig. 2).

**Table 2:** Univariable and multivariable analysis of factors associated with the occurrence of psychological disorders after breast cancer surgery

Outcome	Variables	Univariable analysis		Multivariable analysis	
		HR (95% CI)	*P-*v*alue*	HR (95% CI)	*P-*v*alue*
Overall psychological disorder	Age		< 0.0001		< 0.0001
	40–49 vs. < 40 years	1.022 (0.921, 1.135)	0.6803	1.021 (0.920, 1.134)	0.6918
	50–59 vs. < 40 years	1.201 (1.081, 1.333)	0.0006	1.190 (1.072, 1.322)	0.0011
	≥ 60 years vs. < 40 years	1.091 (0.979, 1.216)	0.1164	1.079 (0.967, 1.203)	0.1735
	Insurance type, Medical Aid vs. Health insurance	1.185 (1.005, 1.396)	0.0429	1.141 (0.967, 1.346)	0.1177
	Axillary surgery, ALND vs SLNBx	1.305 (1.227, 1.388)	< 0.0001	1.337 (1.255, 1.424)	< 0.0001
	Surgery type, TM vs. PM	0.942 (0.887, 1.000)	0.0515	0.889 (0.836, 0.946	0.0002
Depression	Age		0.3895		0.3175
	40–49 vs. < 40 years	0.970 (0.811, 1.160)	0.7376	0.970 (0.811, 1.161)	0.7413
	50–59 vs. < 40 years	1.039 (0.867, 1.245)	0.6814	1.036 (0.864, 1.242)	0.7007
	≥ 60 years vs. < 40 years	0.920 (0.761, 1.112)	0.3895	0.908 (0.750, 1.097)	0.3167
	Insurance type, Medical Aid vs. Health insurance	1.422 (1.083, 1.865)	0.0112	1.420 (1.080, 1.867)	0.0121
	Axillary surgery, ALND vs SLNBx	1.131 (1.011, 1.265)	0.0312	1.124 (1.002, 1.262)	0.0462
	Surgery type, TM vs. PM	1.019 (0.916, 1.133)	0.7308	0.989 (0.887, 1.103)	0.8417
Anxiety Disorder	Age		0.0071		0.0176
	40–49 vs. < 40 years	0.943 (0.805, 1.104)	0.4661	0.946 (0.808, 1.108)	0.4907
	50–59 vs. < 40 years	1.061 (0.906, 1.244)	0.4612	1.055 (0.900, 1.237)	0.5058
	≥ 60 years vs. < 40 years	1.149 (0.978, 1.350)	0.0914	1.134 (0.965, 1.333)	0.1276
	Insurance type, Medical Aid vs. Health insurance	1.323 (1.042, 1.679)	0.0216	1.218 (0.958, 1.549)	0.1079
	Axillary surgery, ALND vs SLNBx	1.440 (1.313, 1.579)	< 0.0001	1.459 (1.327, 1.605)	< 0.0001
	Surgery type, TM vs. PM	0.992 (0.906, 1.087)	0.8678	0.911 (0.829, 1.001)	0.0515
Insomnia	Age		< 0.0001		< 0.0001
	40–49 vs. < 40 years	1.149 (0.993, 1.330)	0.0622	1.148 (0.992, 1.329)	0.0642
	50–59 vs. < 40 years	1.435 (1.241, 1.659)	< 0.0001	1.426 (1.233, 1.649)	< 0.0001
	≥ 60 years vs. < 40 years	1.157 (0.994, 1.347)	0.0603	1.155 (0.991, 1.345)	0.0644
	Insurance type, Medical Aid vs. Health insurance	0.899 (0.701, 1.152)	0.3985	0.885 (0.689, 1.135)	0.3349
	Axillary surgery, ALND vs SLNBx	1.203 (1.107, 1.307)	< 0.0001	1.238 (1.137, 1.349)	< 0.0001
	Surgery type, TM vs. PM	0.926 (0.854, 1.003)	0.0600	0.897 (0.825, 0.974)	0.0100

*TM* total mastectomy, *PM* partial mastectomy, *SLNBx*, sentinel lymph node biopsy, *ALND*, axillary lymph node dissection

Multivariable analysis showed that the rates of overall psychological disorders (HR 1.190, *p* = 0.0011) and insomnia (HR 1.426, *p* < 0.0001) were higher in patients aged 50–59 years than in those aged younger than 40 years. ALND was associated with higher rates of overall psychological disorders (HR 1.337 *p* < 0.0001), depression (HR 1.124 *p *= 0.0462), anxiety (HR 1.459, *p *< 0.0001) and insomnia (HR 1.238, *p* < 0.0001). Rates of overall psychological disorders (HR 0.889, *p* = 0.0002) and insomnia (HR 0.897, *p* = 0.01) were significantly lower in patients who underwent TM than PM (Table 2). Rates of overall psychological disorders (HR 0.837, *p* = 0.0001) and insomnia (HR 0.831, *p* = 0.0021) were also significantly lower in patients who underwent TM plus RS than in those who underwent PM. Compared with patients who underwent TM without RS, those who underwent TM plus RS tended to have lower rates of overall psychological disorders (HR 0.902 *p* = 0.0513) and insomnia (HR 0.878, *p* = 0.0675) (Table 3).

**Table 3:** Comparison of surgery type in the occurrence of psychological disorder after breast cancer surgery

Outcome	Comparisons	Univariable analysis		Multivariable analysis		Risk
		HR (95% CI)	*P-*value	HR (95% CI)	*P-*value	
Overall psychological disorder	TM-RS(−) vs. PM	1.014 (0.944, 1.089)	0.7096	0.928 (0.861, 1.000)	0.0498	TM-RS(+) = TM-RS(−) < PM
	TM-RS(+) vs. PM	0.847 (0.778, 0.923)	0.0001	0.837 (0.767, 0.914)	0.0001	
	TM-RS(+) vs. TM-RS(−)	0.836 (0.757, 0.923)	0.0004	0.902 (0.814, 1.001)	0.0513	
Depression	TM-RS(−) vs. PM	1.008 (0.886, 1.147)	0.8998	0.969 (0.846, 1.109)	0.6432	TM-RS (+) = TM-RS(−) = PM
	TM-RS(+) vs. PM	1.033 (0.895, 1.193)	0.6576	1.015 (0.876, 1.176)	0.8396	
	TM-RS(+) vs. TM-RS(−)	1.025 (0.864, 1.215)	0.7802	1.048 (0.878, 1.252)	0.6036	
Anxiety disorder	TM-RS(−) vs. PM	1.064 (0.955, 1.186)	0.2573	0.927 (0.828, 1.038)	0.1889	TM-RS(+) = TM-RS(−) = PM
	TM-RS(+) vs. PM	0.896 (0.788, 1.020)	0.0967	0.888 (0.778, 1.014)	0.0785	
	TM-RS(+) vs. TM-RS(−)	0.842 (0.725, 0.978)	0.0243	0.958 (0.820, 1.120)	0.5918	
Insomnia	TM-RS(−) vs. PM	0.996 (0.905, 1.096)	0.9290	0.946 (0.856, 1.046)	0.2809	TM-RS(+) < PM@TM-RS(−) = PM@TM-RS(+) = TM-RS(−)
	TM-RS(+) vs. PM	0.832 (0.741, 0.934)	0.0018	0.831 (0.739, 0.935)	0.0021	
	TM-RS(+) vs. TM-RS(−)	0.835 (0.731, 0.955)	0.0086	0.878 (0.764, 1.009)	0.0675	

*TM* total mastectomy, *RS* reconstructive surgery, *PM* partial mastectomy

### Subgroup analysis by age

Patients were dichotomized by age, into a younger (age < 50 years) and an older (age ≥ 50 years) group. Evaluation of younger patients showed that, compared with PM, TM significantly reduced the rates of overall psychological disorders (HR 0.828, *p* = 0.0001), especially of insomnia (HR 0.842, *p* = 0.0086) (Table 4) Moreover, reconstruction after TM had psychological benefits, as patients who underwent TM plus RS had significantly lower rates of overall psychological disorders (HR 0.806, *p* = 0.0002) and insomnia (HR 0.771, *p* = 0.0011) than those who underwent PM. Compared with patients who underwent TM alone, those who underwent TM plus RS had a significantly lower rate of insomnia (HR 0.805, *p* = 0.0378) (Supp. Table 1).

**Table 4:** Multivariable analysis of factors associated with the occurrence of psychological disorders after breast cancer surgery in patients subgrouped by age

Outcome	Variables	Age < 50		Age ≥ 50	
		HR (95% CI)	*P*-value	HR (95% CI)	*P*-value
Overall psychological disorder	Medical Aid vs.Health insurance	1.344 (1.012, 1.785)	0.0414	1.032 (0.843, 1.265)	0.7574
	ALND vs. SLNBx	1.366 (1.239, 1.505)	< 0.0001	1.316 (1.211, 1.430)	< 0.0001
	TM vs PM	0.828 (0.754, 0.910)	0.0001	0.936 (0.862, 1.015)	0.1115
Depression	Medical Aid vs.Health insurance	1.457 (0.899, 2.361)	0.1262	1.339 (0.960, 1.866)	0.0851
	ALND vs. SLNBx	0.973 (0.814, 1.163)	0.7649	1.256 (1.079, 1.461)	0.0033
	TM vs PM	0.902 (0.767, 1.062)	0.2177	1.063 (0.918, 1.231)	0.4144
Anxiety Disorder	Medical Aid vs.Health insurance	0.962 (0.586, 1.579)	0.8779	1.350 (1.026, 1.778)	0.0323
	ALND vs. SLNBx	1.644 (1.422, 1.901)	< 0.0001	1.339 (1.181, 1.519)	< 0.0001
	TM vs PM	0.890 (0.771, 1.027)	0.1118	0.927 (0.819, 1.050)	0.2321
Insomnia	Medical Aid vs.Health insurance	1.288 (0.873, 1.902)	0.2025	0.696 (0.504, 0.962)	0.0581
	ALND vs. SLNBx	1.249 (1.093, 1.427)	0.0011	1.229 (1.099, 1.375)	0.0503
	TM vs PM	0.842 (0.741, 0.957)	0.0086	0.932 (0.836, 1.039)	0.2050

*TM* total mastectomy, *PM* partial mastectomy, *SLNBx* sentinel lymph node biopsy, *ALND* axillary lymph node dissection

**Fig. 3:**
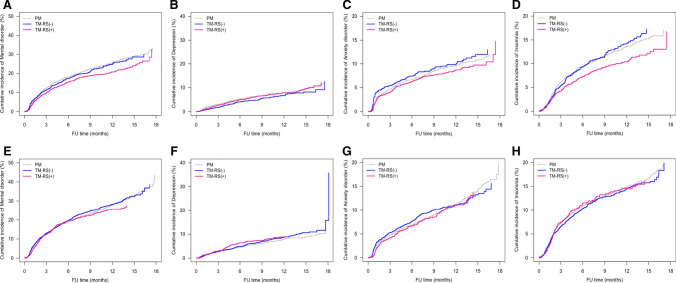
Kaplan–Meier analysis of the incidence of (**a**, **e**) overall psychological disorders, **b**, **f** depression, **c**, **g** anxiety, and **d**, **h** insomnia in patients aged (**a**–**d**) < 50 years and (E–H) ≥ 50 years who underwent TM plus RS, TM alone, and PM alone.

Evaluation of older patients (age ≥ 50 years) showed that type of breast surgery did not affect the incidence of first onset psychological disorders (Fig. 3).

### Subgroup analysis by axillary surgery type

Because this study found that ALND was the most potent variable associated with an increased incidence of overall psychological disorders after breast cancer surgery, patients were subgrouped by type of axillary surgery. ALND did not affect the incidence of psychological disorders, regardless of the type of breast surgery or reconstruction. In patients who underwent SLNBx, TM was associated with significant reductions in the rates of overall psychological disorders (HR 0.843, *p* < 0.0001), anxiety (HR 0.828, *p* = 0.0026), and insomnia (HR 0.876, *p* = 0.0128) compared with PM. TM plus RS was also associated with significantly reduced rates of overall psychological disorders (HR 0.785, *p* < 0.0001), anxiety (HR 0.823 *p* = 0.0236) and insomnia (HR 0.777, *p* = 0.0007) compared with PM (Table 5). The incidence of insomnia was also significantly lower in patients who underwent TM plus RS than in those who underwent TM alone (HR 0.797 *p* = 0.0152) in only SLNBx group (Supp. Table 2).

**Table 5:** Multivariable analysis of factors associated with the occurrence of psychological disorders after breast cancer surgery in patients subgrouped by type of axillary surgery (ALND vs. SLNBX)

Outcome	Variables	ALND		SLNBx	
		HR (95% CI)	*P-*value	HR (95% CI)	*P-*value
Overall psychological disorder	Age		0.2142		< 0.0001
	40–49 vs. < 40 years	1.011 (0.843, 1.214)	0.9027	1.025 (0.902, 1.165)	0.7016
	50–59 vs. < 40 years	1.122 (0.934, 1.347)	0.2196	1.224 (1.077, 1.391)	0.0020
	≥ 60 years vs. < 40 years	1.134 (0.941, 1.366)	0.1876	1.049 (0.917, 1.199)	0.4882
	Insurance type Medical Aid vs. Health insurance				
		1.058 (0.831, 1.348)	0.6456	1.203 (0.959, 1.509)	0.1101
	Surgery type, TM vs PM	0.969 (0.875, 1.073)	0.5461	0.843 (0.779, 0.912)	< 0.0001
Depression	Age		0.0413		0.0068
	40–49 vs. < 40 years	0.808 (0.580, 1.126)	0.2073	1.033 (0.834, 1.279)	0.7679
	50–59 vs. < 40 years	0.979 (0.705, 1.361)	0.9003	1.060 (0.853, 1.317)	0.6018
	≥ 60 years vs. < 40 years	1.159 (0.835, 1.610)	0.3776	0.793 (0.627, 1.002)	0.0516
	*Insurance type*				
	Medical Aid vs. Health insurance	1.325 (0.888, 1.978)	0.1680	1.429 (0.981, 2.083)	0.0630
	Surgery type, TM vs PM	1.082 (0.894, 1.309)	0.4202	0.945 (0.825, 1.081)	0.4103
Anxiety disorder	Age		0.5191		0.0029
	40–49 vs. < 40 years	1.153 (0.878, 1.513)	0.3054	0.852 (0.702, 1.035)	0.1073
	50–59 vs. < 40 years	1.079 (0.817, 1.424)	0.5934	1.044 (0.861, 1.267)	0.6605
	≥ 60 years vs. < 40 years	1.204 (0.910, 1.592)	0.1934	1.097 (0.899, 1.337)	0.3612
	Insurance type				
	Medical Aid vs. Health insurance	0.966 (0.668, 1.397)	0.8556	1.492 (1.088, 2.046)	0.0130
	Surgery type, TM vs PM	1.043 (0.897, 1.214)	0.5832	0.828 (0.732, 0.937)	0.0026
Insomnia	Age		0.0609		< 0.0001
	40–49 vs. < 40 years	1.107 (0.856, 1.433)	0.4381	1.167 (0.977, 1.393)	0.0882
	50–59 vs. < 40 years	1.340 (1.037, 1.731)	0.0253	1.467 (1.230, 1.750)	< 0.0001
	≥ 60 years vs. < 40 years	1.171 (0.898, 1.527)	0.2440	1.146 (0.951, 1.380)	0.1523
	Insurance type				
	Medical Aid vs. Health insurance	0.975 (0.690, 1.378)	0.8847	0.794 (0.553, 1.139)	0.2103
	Surgery type, TM vs PM	0.934 (0.813, 1.074)	0.3402	0.876 (0.790, 0.972)	0.0128

## Discussion

This study demonstrated that the rates of overall psychological disorders and insomnia were significantly lower in patients who underwent TM than PM. Compared with PM alone, reconstruction after TM was significantly associated with reduced rates of overall psychological disorders and insomnia in patients aged < 50 years and in those who underwent SLNBx.

Although most studies have shown that the rate of overall psychological disorders is lower in patients who underwent PM than TM [12, 13, 15], the present study found that this rate was significantly lower in patients who underwent TM (HR 0.918, *p* = 0.007). This finding is in agreement with studies showing that psychological disorders are more frequent after PM than TM [14, 15]. Although PM, also called breast conserving surgery, is regarded as less disfiguring than other types of surgery, esthetic outcomes of PM vary widely, with most women reporting breast asymmetry [21]. Most patients who undergo PM also require radiation, which can lead to poor esthetic outcomes. These unsatisfactory cosmetic outcomes may result in a higher prevalence of overall psychological disorders. Healthcare professionals should therefore not assume that PM confers psychological benefit compared with TM alone. Moreover, patients who undergo PM may be more concerned about remaining tumors, resulting in greater psychological pressure. It is therefore important to inform breast cancer patients that, like TM, PM is also radical surgery, with the two having similar long term survival rates.

Multivariable analysis showed that ALND was the most significant factor associated with increased rates of overall psychological disorders, depression, anxiety and insomnia. Patients who undergo ALND have more advanced stage disease than patients who undergo SLNBx. During the first year after surgery, fear of being diagnosed with an advanced stage tumor may have greater psychological impact than cosmetic satisfaction. Subgroup analysis of patients who underwent ALND found that type of breast cancer surgery had no impact on the development of psychological disorders.

Comparison of the rates of psychological disorders in patients who underwent PM, TM without RS, and TM with RS, found that the rates of overall psychological disorders and insomnia were lowest in patients who underwent TM with RS. RS tended to benefit patients who underwent TM, lowering the rates of overall psychological disorders and insomnia. Subgroup analysis showed that, compared with PM alone, RS after TM was significantly associated with lower rates of insomnia in patients aged < 50 years and in those who underwent SLNBx. Of the 4056 patients who underwent RS after TM, 4030 (99.36%) underwent immediate reconstruction. Thus, removal of the cancer by TM, followed by immediate reconstruction resulting in cosmetic satisfaction may have psychological advantages when compared with PM during the early postoperative period.

RS after mastectomy may benefit breast cancer patients by improving psychological damage resulting from mastectomy. Similarly, a meta-analysis showed that RS could significantly associated with reduced rates of anxiety (RR = 0.62, 95% CI 0.47–0.82, *p* = 0.0006) and depression (RR = 0.54, 95% CI 0.32–0.93, *p* = 0.02) when compared with mastectomy alone [22]. However, Beck Depression Inventory-13 (BDI-13) scores were found to be similar in breast cancer patients who underwent mastectomy alone and reconstruction after mastectomy [18]. In addition, rates of depressive symptoms at a mean 1 year after surgery did not differ significantly in breast cancer patients who underwent TM, breast conserving surgery and RS [23]. Insomnia rates are up to three-fold higher in patients with cancer than in the general population, with the highest rates observed in patients with breast cancer [5].

Multivariable analysis showed that rates of overall psychological disorders (HR 1.177, *p* = 0.0025) and insomnia (HR 1.406, *p* < 0.0001) were significantly higher in patients aged ≥ 50 years than in patients aged < 50 years. In the older group, type of breast surgery did not affect the incidence of first onset psychological disorders after breast cancer surgery. In the younger group, however, RS was significantly associated with a reduced prevalence of overall psychological disorders and insomnia compared with PM.

The present study had several limitations. Because HIRA data consist only of diagnostic codes and demographic information, clinical data such as cancer stage at diagnosis, medical comorbidities; sociodemographic factors, including education level and marital status; and psychosocial factors were unavailable. Future studies combining HIRA claims data with clinical datasets are necessary. Additionally, the use of ICD codes may have resulted in patient misdiagnosis. However, most previous studies were based on self-reported questionnaires, including BDI scores, rather than diagnostic instruments. Drugs for psychological disorders could not be prescribed to patients without the doctor entering the exact diagnostic into the Korean insurance system. Therefore, a medical diagnosis based on an ICD code would be more accurate than survey or other subjective data used in other studies. Additionally, the present study did not assess the impact of additional treatments, such as chemotherapy, radiation, or endocrine therapy, on psychological disorders in these patients. Psychological and emotional functioning were shown to improve in most breast cancer patients during the postoperative period [4]. Because the mean postoperative follow-up time in the present study was relatively short (< 1 year), types of breast cancer surgery may have different long-term effects on psychological disorders.

Nonetheless, the results of this study are meaningful, in that this was the first study to assess the impact of breast surgery type on initial onset of psychological disorders after breast cancer surgery using accurate and comprehensive big data. The occurrence of anxiety, depression and insomnia after immediate postoperative period was < 10% for each.

In conclusion, the present study showed that the incidence of overall psychological disorders and insomnia were higher in patients who underwent PM than TM. Compared with PM, RS after TM was significantly associated with a reduced incidence of overall psychological disorders and insomnia in younger patients (age < 50 years) and those who underwent SLNBx when assessed at a postoperative follow-up of < 1 year. The surgeon should inform their patients strongly that TM is not safer than PM with the two having similar longer survival.

## Supplementary Information

Below is the link to the electronic supplementary material.

Supplementary file1 (DOCX 19 KB)

Supplementary file2 (PPTX 47 KB)

## Data Availability

All relevant data are within the paper and its supporting information.
