# Correction to: Psychological Impact of Type of Breast Cancer Surgery: A National Cohort Study

**DOI:** 10.1007/s00268-022-06607-9

**Published:** 2022-05-25

**Authors:** Soo kyung Ahn, Sohee Oh, Jongjin Kim, Jung-Seok Choi, Ki-Tae Hwang

**Affiliations:** 1 Department of Surgery, Kangnam Sacred Heart Hospital, Hallym University College of Medicine, 1 Shingil-ro, Youngdeungpo-ku, Seoul, 07441 Republic of Korea; 2 Medical Research Collaborating Center, Seoul Metropolitan Government Seoul National University Boramae Medical Center, Seoul, 07061 Republic of Korea; 3 Department of Surgery, Seoul Metropolitan Government Seoul National University Boramae Medical Center, Seoul, Republic of Korea; 4 Department of Psychiatry, SMC, Seoul, Republic of Korea; 5 Department of Surgery, Seoul National University College of Medicine, Seoul Metropolitan Government Seoul National University Boramae Medical Center, 39, Boramae-Gil, Dongjak-gu, Seoul, 07061 Republic of Korea

## Correction to: World J Surg 10.1007/s00268-022-06585-y

The following note was missing from the original online version of this article:

Soo kyung Ahn and Sohee Oh contributed equally to this work.

The original article was corrected.

