# Supplementary material for: Psychological Impact of Type of Breast Cancer Surgery: A National Cohort Study
Source: World J Surg. 2022 May 10;46(9):2224–33. doi: 10.1007/s00268-022-06585-y (PMC9334396; doi:10.1007/s00268-022-06585-y)
Supplement: Supplementary file 1 — Supplementary file1 (DOCX 19 KB) [file 268_2022_6585_MOESM1_ESM.docx]

Supporting Table 1. Comparison of surgery type in the occurrence of psychological disorder after breast cancer surgery subgrouped by age

| **Outcome** | **Comparisons** | **Age<50** | | **Risk** |  | **Age≥50** | | **Risk** |
| --- | --- | --- | --- | --- | --- | --- | --- | --- |
|  |  | **HR (95% CI)** | **P-value** |  |  | **HR (95% CI)** | **P-value** |  |
| Overall psychological disorder | TM-RS(-) vs. PM | 0.862 (0.757,0.983) | 0.0264 | TM-RS(+)=TM-RS(-) < PM |  | 0.951 (0.868, 1.041) | 0.2750 | TM-RS(+) = TM-RS(-) = PM |
|  | TM-RS(+) vs. PM | 0.806 (0.721,0.903) | 0.0002 |  |  | 0.896 (0.781, 1.028) | 0.1180 |  |
|  | TM-RS(+) vs. TM-RS(-) | 0.935 (0.804,1.088) | 0.3872 |  |  | 0.943 (0.812, 1.095) | 0.4394 |  |
| Depression | TM-RS(-) vs. PM | 0.802 (0.630, 1.021) | 0.0729 | TM-RS(+) = TM-RS(-) = PM |  | 1.042 (0.884, 1.228) | 0.6239 | TM-RS(+) = TM-RS(-) = PM |
|  | TM-RS(+) vs. PM | 0.965 (0.800, 1.163) | 0.7050 |  |  | 1.121 (0.886, 1.418) | 0.3427 |  |
|  | TM-RS(+) vs. TM-RS(-) | 1.203 (0.918, 1.577) | 0.1797 |  |  | 1.076 (0.832, 1.390) | 0.5779 |  |
| Anxiety Disorder | TM-RS(-) vs. PM | 0.936 (0.770, 1.138) | 0.5085 | TM-RS(+) = TM-RS(-) = PM |  | 0.932 (0.812, 1.070) | 0.3152 | TM-RS(+) = TM-RS(-) = PM |
|  | TM-RS(+) vs. PM | 0.860 (0.725, 1.021) | 0.0847 |  |  | 0.915 (0.743, 1.125) | 0.3980 |  |
|  | TM-RS(+) vs. TM-RS(-) | 0.919 (0.733, 1.151) | 0.4617 |  |  | 0.982 (0.783, 1.231) | 0.8721 |  |
| Insomnia | TM-RS(-) vs. PM | 0.957 (0.805, 1.138) | 0.6193 | TM-RS(+) < TM-RS(-) = PM |  | 0.920 (0.814, 1.040) | 0.1837 | TM-RS(+) = TM-RS(-) = PM |
|  | TM-RS(+) vs. PM | 0.771 (0.660, 0.901) | 0.0011 |  |  | 0.962 (0.806, 1.149) | 0.6701 |  |
|  | TM-RS(+) vs. TM-RS(-) | 0.805 (0.657, 0.988) | 0.0378 |  |  | 1.045 (0.860, 1.271) | 0.6552 |  |

Abbreviations: TM, total mastectomy; RS, reconstructive surgery; PM, partial mastectomy

Supporting Table 2. Comparison of surgery type in the occurrence of psychological disorder after breast cancer surgery subgrouped by type of axillary surgery (ALND vs. SLNBX)

| **Outcome** | **Comparisons** | **ALND** | | **Risk** |  | **SLNBx** | | **Risk** |
| --- | --- | --- | --- | --- | --- | --- | --- | --- |
|  |  | **HR (95% CI)** | **P-value** |  |  | **HR (95% CI)** | **P-value** |  |
| Overall psychological disorder | TM-RS(-) vs. PM | 0.977 (0.872, 1.094) | 0.6829 | TM-RS(+) = TM-RS(-) = PM |  | 0.899 (0.812, 0.995) | 0.0399 | TM-RS(+) = TM-RS(-) < PM |
|  | TM-RS(+) vs. PM | 0.952 (0.820, 1.106) | 0.5215 |  |  | 0.785 (0.704, 0.875) | <0.0001 |  |
|  | TM-RS(+) vs. TM-RS(-) | 0.975 (0.833, 1.142) | 0.7541 |  |  | 0.873 (0.760, 1.003) | 0.0553 |  |
| Depression | TM-RS(-) vs. PM | 1.020 (0.825, 1.262) | 0.8525 | TM-RS(+) = TM-RS(-) = PM |  | 0.943 (0.787, 1.128) | 0.5193 | TM-RS(+) = TM-RS(-) = PM |
|  | TM-RS(+) vs. PM | 1.227 (0.939, 1.604) | 0.1344 |  |  | 0.947 (0.793, 1.131) | 0.5476 |  |
|  | TM-RS(+) vs. TM-RS(-) | 1.203 (0.907, 1.594) | 0.1998 |  |  | 1.005 (0.795, 1.269) | 0.9679 |  |
| Anxiety Disorder | TM-RS(-) vs. PM | 1.057 (0.894, 1.249) | 0.5173 | TM-RS(+) = TM-RS(-) = PM |  | 0.832 (0.709, 0.977) | 0.0247 | TM-RS(+) = TM-RS(-) < PM |
|  | TM-RS(+) vs. PM | 1.015 (0.816, 1.262) | 0.8967 |  |  | 0.823 (0.696, 0.974) | 0.0236 |  |
|  | TM-RS(+) vs. TM-RS(-) | 0.960 (0.764, 1.206) | 0.7260 |  |  | 0.989 (0.796, 1.229) | 0.9223 |  |
| Insomnia | TM-RS(-) vs. PM | 0.927 (0.794, 1.083) | 0.3406 | TM-RS(+) = TM-RS(-) = PM |  | 0.975 (0.854, 1.113) | 0.7064 | TM-RS(+) < TM-RS(-) = PM |
|  | TM-RS(+) vs. PM | 0.950 (0.776, 1.163) | 0.6198 |  |  | 0.777 (0.671, 0.899) | 0.0007 |  |
|  | TM-RS(+) vs. TM-RS(-) | 1.025 (0.826, 1.271) | 0.8250 |  |  | 0.797 (0.663, 0.957) | 0.0152 |  |

Abbreviations: TM, total mastectomy; RS, reconstructive surgery; PM, partial mastectomy
