# Supplementary material for: Psychological Impact of Type of Breast Cancer Surgery: A National Cohort Study
Source: World J Surg. 2022 May 10;46(9):2224–33. doi: 10.1007/s00268-022-06585-y (PMC9334396; doi:10.1007/s00268-022-06585-y)
Supplement: Supplementary file 2 — Supplementary file2 (PPTX 47 KB) [file 268_2022_6585_MOESM2_ESM.pptx]

## Slide 1
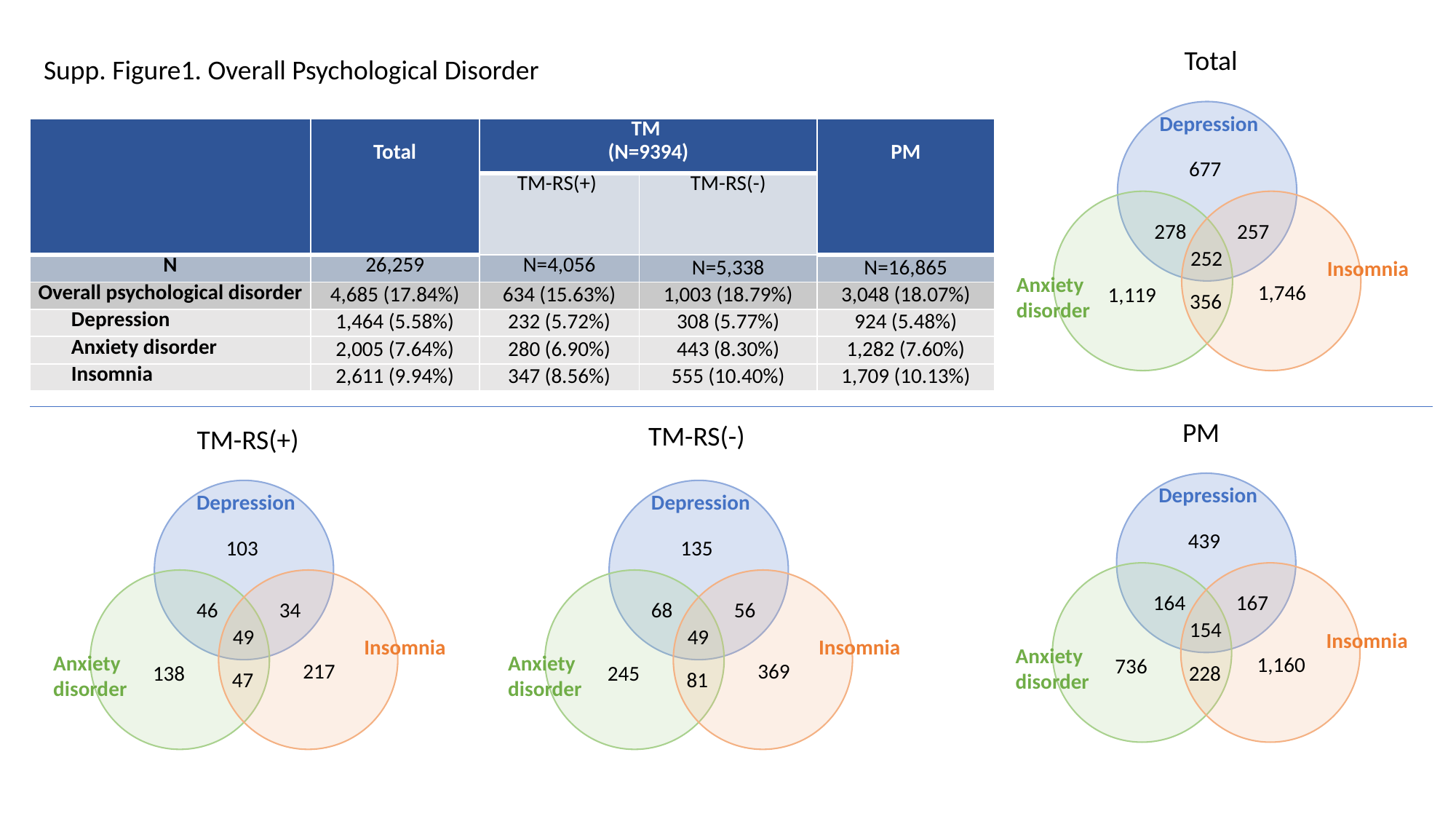

Total
Supp. Figure1. Overall Psychological Disorder
Depression
677
278
257
252
Insomnia
Anxiety
disorder
1,746
1,119
356
| | Total | TM (N=9394) | | PM |
| --- | --- | --- | --- | --- |
| | | TM-RS(+) | TM-RS(-) | |
| N | 26,259 | N=4,056 | N=5,338 | N=16,865 |
| Overall psychological disorder | 4,685 (17.84%) | 634 (15.63%) | 1,003 (18.79%) | 3,048 (18.07%) |
| Depression | 1,464 (5.58%) | 232 (5.72%) | 308 (5.77%) | 924 (5.48%) |
| Anxiety disorder | 2,005 (7.64%) | 280 (6.90%) | 443 (8.30%) | 1,282 (7.60%) |
| Insomnia | 2,611 (9.94%) | 347 (8.56%) | 555 (10.40%) | 1,709 (10.13%) |
PM
TM-RS(-)
TM-RS(+)
Depression
439
164
167
154
Insomnia
Anxiety
disorder
1,160
736
228
Depression
103
46
34
49
Insomnia
Anxiety
disorder
217
138
47
Depression
135
68
56
49
Insomnia
Anxiety
disorder
369
245
81
